# Supplementary material for: Plasmon-Enhanced Perovskite Solar Cells Based on Inkjet-Printed Au Nanoparticles Embedded into TiO2 Microdot Arrays
Source: Nanomaterials (Basel). 2023 Sep 29;13(19):2675. doi: 10.3390/nano13192675 (PMC10574114; doi:10.3390/nano13192675)
Supplement: Supplementary file 1 [file nanomaterials-13-02675-s001.zip › nanomaterials-2589796-supplementary.pdf]

nanomaterials

Supplementary information

Plasmon-Enhanced Perovskite Solar Cells Based on Inkjet-Printed Au Nanoparticles Embedded into TiO_2 Microdot Arrays

Sofia Rubtsov [1], Albina Musin [2], Viktor Danchuk [1], Mykola Shatalov [1], Neena Prasad [1], Michael Zinigrad [1] and Lena Yadgarov [1,*]

[1] Department of Chemical Engineering, Biotechnology and Materials, Faculty of Engineering, Ariel University, Ariel 4076414, Israel; sofiar@ariel.ac.il (S.R.); viktorde@ariel.ac.il (V.D.); mykolash@ariel.ac.il (M.S.); neenaphy@gmail.com (N.P.); zinigradm@ariel.ac.il (M.Z.)
[2] Physics Department, Faculty of Natural Sciences, Ariel University, Ariel 4076414, Israel; albinam@ariel.ac.il
* Correspondence: lenay@ariel.ac.il

1. Preparation of Ink

Two types of ink were used for MDA (microdots array) printing on the ETL layer of the PSC: one ink was with titanium dioxide nanoparticles (TiO_2 NPs), and the other contained TiO_2 NPs with Au NPs. The TiO_2 NPs were synthesized via the sol-gel method [1], using titanium isopropoxide ($Ti[OCH(CH_3)_2]_4$, 99.995%, Alfa Aesar, UK), nitric acid (HNO_3 70%, Daejung,) and isopropanol (C_3H_8O Carlo Erba). Ink with gold complex nanoparticles (AuCNP) was synthesized by a modified polyol method with TiO_2 support. The colloidal solution containing 0.125 g of TNP xerogel, 6 mL ethylene glycol (EG) (Daejung), and 2 mL pure water (Milli-Q® IQ 7003, Merck, Germany) was heated to 150 °C. The second solution of 0.0677 g hydrogen tetrachloroaurate (III) trihydrate ($HAuCl_4{\cdot}3H_2O$ 99.99%, Alfa Aesar) in 2 ml DW and 1.2 µl of surfactant Tween 80 (Sigma-Aldrich, Germany) was added into the flask with boiling TiO_2 colloid, and the mixture was boiled for 10 min under stirring (500 rpm). Finally, the resulting dispersion of AuCNP was cooled to room temperature and stored at 8 °C in a lab refrigerator. Before filling a cartridge, the inks were filtered with a 0.22 µm filter (Jet Biofil, China). The characteristics of the suspension are given in the previous article [2].

2. Inkjet Printing Methods

The MDA patterns of TiO_2 and TiO_2-AuNP were deposited on the substrates using a Dimatix Materials DMP 2850 printer (Santa Clara, CA, USA) with a 10 pL cartridge. Each dot was obtained from a single jetted droplet with a drop spacing of about 80 µm. The drop spacing was chosen to be larger than the contact diameter of a single drop on a substrate (35 µm) so that the drops could not merge. The printing was carried out with a jetting frequency of 5 kHz, the voltage on the nozzle was varied in the range of 19 to 25 V, and only one nozzle was used to print the pattern. During printing, the temperature of the substrates was stabilized at 30°C. After printing the patterns, the samples were annealed at 550 °C for 60 min with a 60 sccm flow of dry air (ALIMC-500 ccm, Alicat Scientific, USA) in the Tube Furnace KJ-T1200 (Zhengzhou Kejia Furnace Co., Ltd). The final reduction of Au and formation of TiO_2-AuNP_MDA occurred during the annealing of the samples [2].

3. FTO Etching

FTO coating was etched to receive a desired configuration of conducting electrodes using zinc powder and hydrochloric acid (Alfa Aesar, USA) according to the accepted procedure (see, e.g., https://www.ossila.com/products/fto-glass-unpatterned?variant=21518956481).

4. Substrate Cleaning Protocol

Soda-lime 0215 Corning Glass (Corning, USA) and fluorine-doped tin oxide (FTO) coated glass (Biotain Crystal Co, China, 2.2 mm thickness, 10–15 Ω/sq, 25 mm ×25 mm) were used as substrates for PSC device fabrication. The FTO layer was etched to create electrode contacts for multi-pixel devices. All substrates were cleaned before coating according to the detailed protocol. Glass and etched FTO substrates with the size of 75 mm × 50 mm were sonicated in a bath with Micro-90 cleaning solution (International Products Corporation, USA), diluted with distilled water 1:50, at 80 °C during 10 min in an ultrasonic cleaner (GT Sonic-P3, China). After that, the substrates were rinsed with deionized water to remove the residual of the cleaning solution and then kept in an ultrasonic bath with deionized water for 10 min. Further, the substrates were dried in airflow and sonicated in isopropanol for 10 min at 80 °C. After that, the substrates were sonicated in Milli-Q water, blown with dry nitrogen, immersed in boiling Milli-Q water for 5 s, dried in a nitrogen flow, and finally heated in a vacuum drying oven (Faithful Instruments, China) for 20 min at 120 °C. Clean substrates were stored in a desiccator under a vacuum. Before use, the glasses were cleaved into squares of 25 × 25 mm and rinsed in the spin-coater (WS-650HZ-23NPPB, Laurell Technologies Inc, USA) subsequently with 3 mL bi-distilled water, 0.5 mL isopropanol, 3 mL bi-distilled water, 0.5 mL isopropanol, 3 mL bi-distilled water (×2 times) at 4000 rpm, and finally centrifuged at 6000 rpm for 15 sec. After rinsing, glasses were dried in the oven for 20 min at 120°C. Just before the vacuum deposition of ETL, the glass substrates were processed in a vacuum plasma cleaner (MTI Corp. EQ-PCE3, USA) for 2 min at a "High" regime in residual air at 0.18 Torr.

5. ETL Deposition

TiO_2 ETL layers were deposed on FTO substrates with VST TFSP-842 (VST, Israel) magnetron sputter. Before deposition, the vacuum chamber was evacuated by a turbomolecular pump to vacuum no worse than 2.2·× 10^{-6} Torr. The pressure of working gas (Ar) in the chamber was 10 mTorr. The deposition power (RF) applied on the TiO_2 magnetron target (Testbourne LTD, 99.99% purity, 2-inch diameter, 0.125-inch thickness) was 100 W. To remove potential impurities from the surface of the target, the target was preliminarily sputtered with a closed shutter at the above conditions for 20 min. The deposition time was 20 min at room temperature. To ensure uniformity of the coating, the sample table rotated at 10 rpm. The thickness of TiO_2 ETL, defined by the gravimetric method [3], was 60 nm. After the deposition, the substrates were annealed at 550 °C for 60 min under 600 sccm dry airflow in the Tube Furnace.

6. Energy Band Diagram

The following diagram introduces how the electrons are collected by the n-type ETL and the holes by the Spiro-MeOTAD - p-type HTL. These electrons then proceed to the external circuit and reach the p-type layer, where they recombine with the holes. Figure S1 illustrates the band diagram of a perovskite solar cell device with ETL incorporated with TiO_2_ AuNP MDA [4]. Introducing AuNPs improves the charge transport and collection compared to the pure TiO_2. Interestingly, the Au NPs act as the scattering point source and thus can extend the distance over which light travels in perovskite, effectively improving the absorption of photons of the active layer [4].

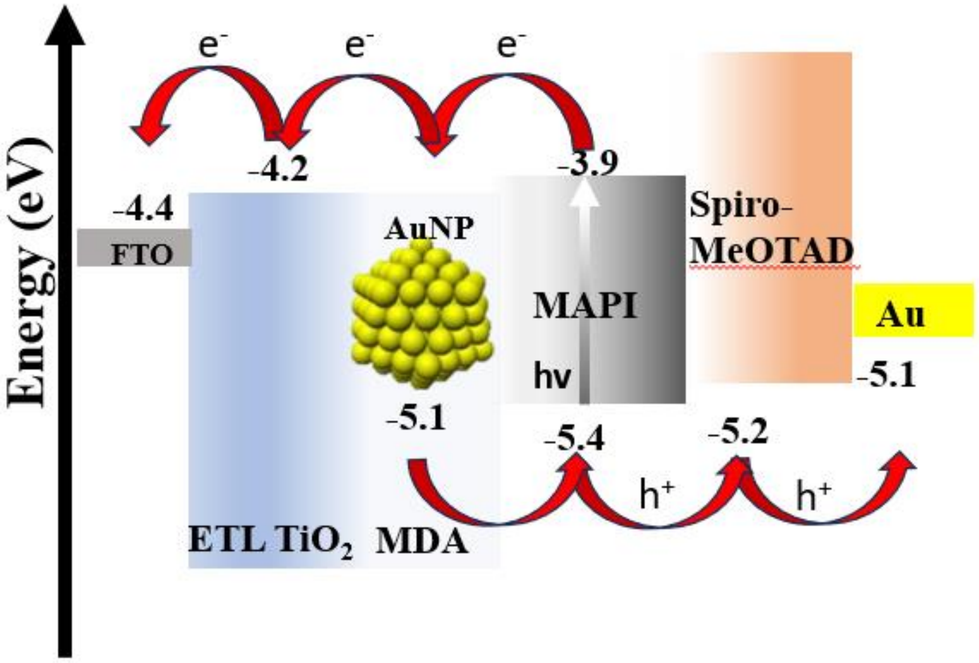

Figure S1. Energy diagram of the plasmon-enhanced TiO_2/AuNP MDA-based perovskite solar cells.

7. FDTD simulation

The schematic representation of a conventional FDTD simulation cell is depicted in Figure S2 within the simulation environment. This cell plays a crucial role in the iterative solution of the discrete Maxwell equations. Within this cell, individual elements or sub-cells can be substituted with parameters such as refractive index (n), conductivity coefficient (σ), electric permittivity (ε), or magnetic permeability (μ) [5]. Each of these elements or sub-cells essentially emulates a portion of a nanoparticle or its immediate surroundings. In the context of this procedure, the optical properties of Au are characterized using the Drude-Lorentz equation.

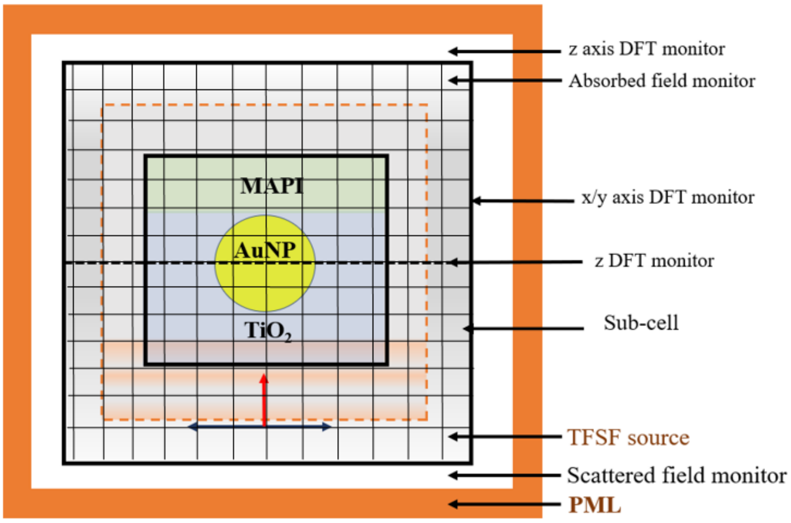

Figure S2. Schematic description of 2D FDTD simulation setup. The cross-section of the NP is located at the center of the simulation region. It is irradiated by a pulse of the "total field scattered field" (TFSF) source, which is confined to the orange-dashed square. The field monitors record the power absorbed/scattered by the NP. The electric field in the region of NP was recorded as well to get the model profiles. Perfectly matched layers (PML) were used as absorbing boundary conditions.

8. AuNP Size Distribution and Thickness of MDA

The size distribution of AuNPs was calculated with "ImageJ" software to be in the range of 25 ± 14 nm and presented in Figure S3.

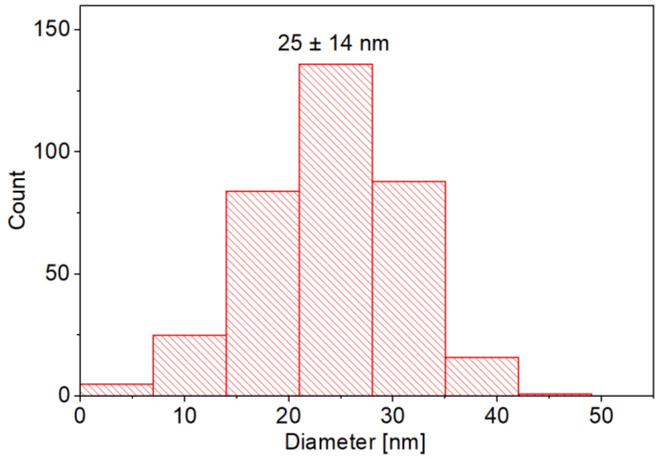

Figure S3. AuNP size distribution from ImageJ software with a modified "particle size" plugin.

9. The Detailed Thickness Calculation of the MDA

The printed solution contains 0.125 g of TNP xerogel, which consists of 75% dry TiO_2 NP, ~0.0875 g of TiO_2 NP weight. Additionally, 0.0677 g of $HAuCl_4{\cdot}3H_2O$ is present, leading to the disposition of 0.0339 grams of Au NPs. Each droplet, dispensed from a single nozzle with a consistent volume of 10 pL, corresponds to a calculated dot volume of 22.44 μm³. The dot has a diameter of 35 μm, and its height can be determined using the formula for the volume of an ellipsoid: $V = \frac{2}{3}\pi r^2 H$, where 'r' represents the radius of the dot, and 'H' represents the thickness of the dot we seek to determine. The dot's height is found to be 35 nm; however, it should be noted that the dot is not perfectly ideal. To achieve greater accuracy, the thickness of the dot is reported with a range of 35 ± 10 nm.

10. TEM of TiO_2_AuNP and SEM Cross-Section of MAPI

Figure S4 shows **A** - are embedded in TiO_2 because of the TiO_2 crystalline planes that cover gold NP, and **B** – SEM cross-section of MAPI deposited on glass, the thickness of the MAPI layer is 390 nm.

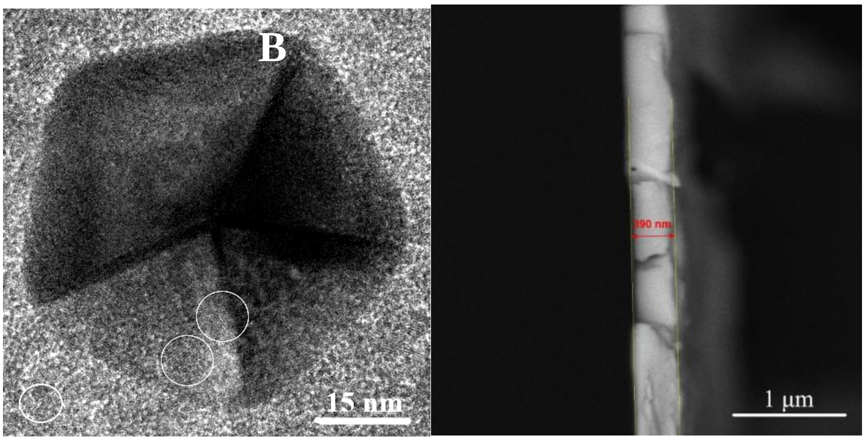

Figure S4. A - TEM micrograph of an Au NP embedded in TiO_2, B – SEM image of MAPI cross-section.

11. Precursors Preparation and PSC Fabrication

The precursor solutions for MAPI and Spiro-MeOTAD were prepared in a glovebox under a nitrogen atmosphere according to the commonly used methods[6,7].

For MAPI, 461 mg of PbI_2 (Sigma-Aldrich, 99.999%, perovskite grade) and 163 mg of methylammonium iodide (Sigma-Aldrich, >99%, anhydrous) were dissolved in 1 ml of a mixture of N,N-dimethyl formamide (Sigma-Aldrich, >99.9, anhydrous) and dimethyl sulfoxide (Sigma-Aldrich, >99.9, anhydrous) 9:1 by volume. The solution was stirred at room temperature by a magnetic stirrer at 550 rpm for 2 h. For the reference PSC, MAPI precursor solution was deposited on the ETL with a single-step spin-coating technique in a nitrogen-filled glove box [6], washed twice with chlorobenzene (500 and 250 μL) as the anti-solvent treatment. The MAPI was annealed on a hot plate at 90 °C for 15 min.

Spiro-MeOTAD solution was prepared by dissolving 250 mg of spiro-MeOTAD (Ossila, UK) in 3.1 mL of chlorobenzene (Sigma-Aldrich, >99.8, anhydrous), with addition of 100 μL of 4-tert-butylpyridine (Acros Organics) and 56 μL of lithium bis(trifluoromethanesulfonyl)imide (Alfa Aesar) stock solution (516 mg in 1 mL of acetonitrile (Acros Organics, 99.9%)). The HTL was deposited on MAPI by spin-coating Spiro-MeOTAD solution at 4000 rpm in the glove box. Finally, the 120 nm Au top electrodes were thermally evaporated on the top of the HTL layer.

For the modified PSCs, an additional layer of TiO_2_MDA or TiO_2-AuNPs_MDA was inkjet printed on the top of the ETL layer and annealed, see Figure S5 of the MAPI surface under printed MDA.

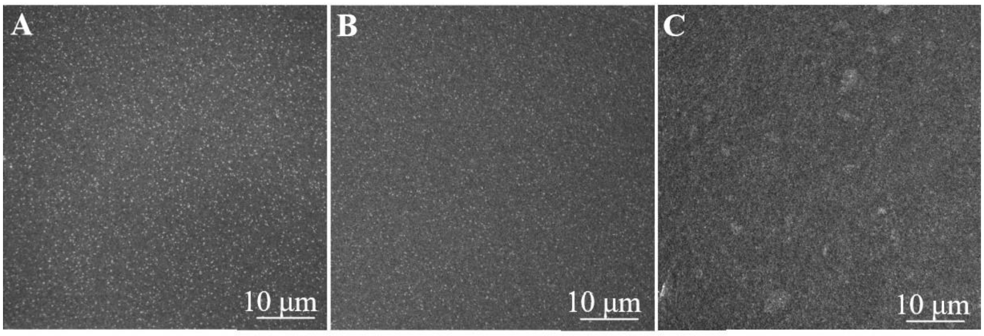

Figure S5. SEM images of MAPI surface on **A** – glass substrates, **B** – glass with TiO_2_MDA, and **C** – glass with Au/TiO_2_MDA.

12. Relationship between the LSPR of Au NPs and the Refractive Index of the Surrounding Media

According to Mie theory, the intensity of light-scattering is related to the metal NPs size, which can be described by Equation (1).

$$\sigma_{\mathrm{scat}} = \frac{8\pi}{3} k^4 a^6 \left| \frac{\varepsilon_{\mathrm{metal}} - \varepsilon_{\mathrm{m}}}{\varepsilon_{\mathrm{metal}} - 2\varepsilon_{\mathrm{m}}} \right|^2 \quad (1)$$

here, σ_{scat} is the scattering cross-section, $k = 2\pi/\lambda$, a is the diameter of the spherical NP, ε_{metal} and ε_m are the dielectric constants of the metal and surrounding medium, respectively.

From Equation (1) we learn that the surrounding medium has a considerable effect on the scattering and the absorbance of the NPs. The LSPR frequency can be fitted by implementation of the Drude model using the following equation: Equation (2) [8,9].

$$\omega_{\mathrm{sp}} = \sqrt{\frac{\omega_p^2}{1+2\varepsilon_{\mathrm{m}}} - \gamma^2} \quad (2)$$

here ω_p is the bulk plasma oscillation frequency associated with the free carriers, ω_{sp} represents the LSPR energy and γ represents the linewidth of the plasmon resonance band. Equation (2) further indicates that the plasmonic resonance is affected by the surrounding medium.

Relationship between the LSPR extinction band of spherical Au NPs and the refractive index of the surrounding media can be also described using the discrete dipole approximation (DDA) theory and the Drude model as shown in the following Equation (3)

$$\lambda = \lambda_p\sqrt{2\varepsilon_m + 1} \cong \sqrt{2}\lambda_p n_m \quad (3)$$

where λ and λ_p are the LSPR wavelengths of NP in dielectric coating and bare NPs, respectively; n_m and ε_m are the refractive index and dielectric constant of the surrounding matrix, respectively ($\varepsilon = n_2$). It is clear from this equation, that the LSPR wavelength of NP in dielectric coating has a linear relationship with the refractive index of the surrounding medium, and the LSPR extinction peak undergoes a red-shift as the refractive index of the dielectric matrix increases [10].

Absorption cross-sections of AuNP, TiO_2, MAPI, and their hybrids were extracted from the FDTD simulations. Not normalized absorption curves are presented in Figure S6. When AuNP is in TiO_2 environment, the intensity of absorption rises, as shown by the black curve of bare AuNP compared to Au Np imbedded in TiO_2.

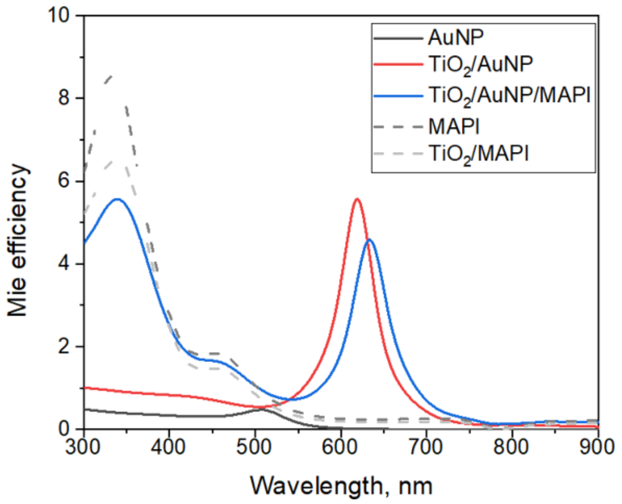

Figure S6. FDTD – simulated not normalized absorption cross-section.

The LSPR of the AuNP suspended in air extinction peak, simulated at FDTD at ~508 nm, see Figure S7.

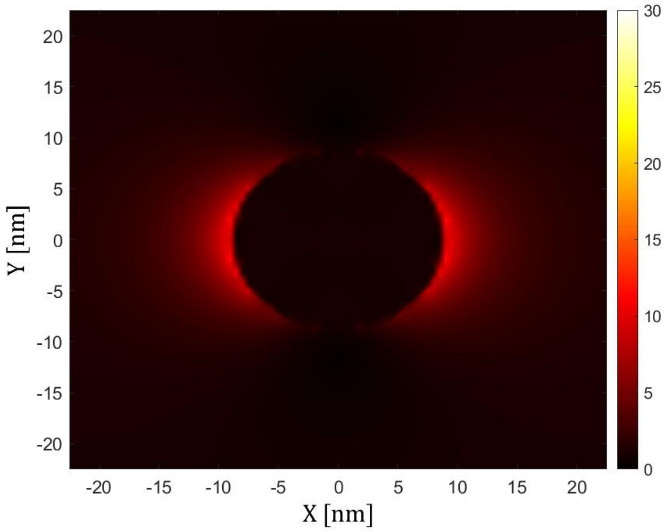

Figure S7. FDTD simulated electromagnetic-intensity distribution for the bare Au NP at λ = 508 nm.

13. Reflectance UV-Vis Spectra

A spectrophotometer Sphere Jasco V-750 was used to measure percentage reflectance of glasses, Figure S8, with MAPI, MAPI/TiO_2_MDA and MAPI/TiO_2-AuNP_MDA samples, bare glass was taken as a baseline. The Equation (4) [11] that was used to calculate the absorbance from reflectance measurements is:

$$A = -\log(I/I_0) = -\log(R) \quad (4)$$

where, A – represents absorption, I – light reflected from a sample, I_0 – the incident energy and R – reflectance.

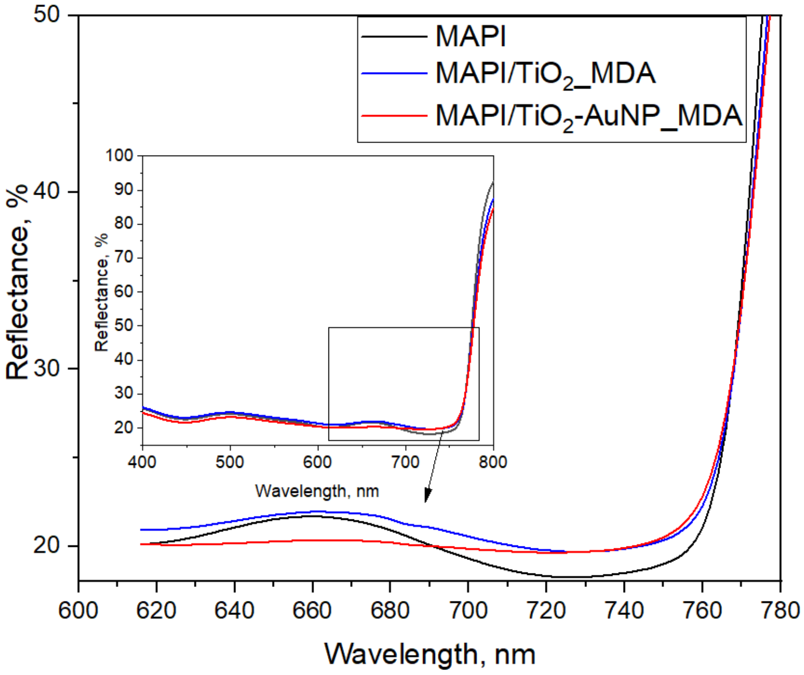

Figure S8. Measured reflectance of glass surfaces covered with MAPI, MAPI/TiO_2_MDA and MAPI/TiO_2-AuNP_MDA.

14. Photovoltaic Parameters of PSCs after One Week of Storage

Table S1 displays SC measurements following one week of storage. Compared to the initial measurements in Table S1, we can observe some parameters degrading over time for all of the samples. However, the J_{SC} parameter for the devices with printed dots has been enhanced, the highest value of TiO_2_MDA sample improved from 20.5 to 22.8 mA/cm^2, while for the TiO_2-AuNP_MDA sample improved from 23.3 to 25.2 mA/cm^2. Table S1 presents a performance comparison of previously reported devices that incorporate AuNPs within PSCs.

The perovskite photovoltaic devices combined with TiO_2 –Au-NPs composite layers achieve higher values of the performance according to articles [4,12–15]. It's crucial to acknowledge that the previously published works primarily include a minimum of 0.3% w/w of AuNPs [12–14]. In contrast, our present work introduces a proof-of-concept setup using only 0.25% w/w of AuNPs, hence our work is in the beginning of the research and concentrations adjustments. And yet, the fabricated device exhibits superior performance of Jsc to the previously reported works.

Table S1. Photovoltaic performance of the champion devices after 7 days of storage in glove box: reference device, with TiO_2_MDA, and with TiO_2-AuNP_MDA.

Samples	J_{SC}, mA/cm^2, ±0.1	V_{OC}, V, ± 0.01	PCE, %, ± 0.3	FF, %, ± 0.1	Reference
Reference device	19.8	0.98	8.9	38.1	Present Work
TiO_2_MDA	22.8	1.03	10	42.7	Present Work
TiO_2-AuNP_MDA 0.25% wt	25.2	0.94	10.2	43.0	Present Work
TiO_x_AuNP (Composite layer) 0.4% wt	19.9	1.08	16.2	76	[12]
Au@ TiO_2 Nanofibers 0.3% wt	20.83	0.986	14.4	70	[4]
AuNP in TiO_2 ETL0.5% wt	17.4	0.76	8	62.4	[13]
Core@ShellAu@TiO_2NPs 0.5% wt	21.2	1.03	16	73.3	[14]
AuNP in TiO_2 ETL 0.2%wt	24.5	1.10	20.3	75.5	[15]

15. Calculation of Band Gap Energy

Figure S9 shows the Kubelka-Munk plot for three samples deposited on glass substrates: MAPI – black curve, MAPI with TiO_2 _MDA – blue curve and MAPI with TiO_2-AuNP_MDA – red curve. The optical absorption coefficient α may be calculated using reflectance data according to the Equation (5) [16]:

$$F(R) = \alpha = \frac{(1-R)^2}{2R} \quad (5)$$

where R is the reflectance. Optical parameters are related via the Kubelka-Munk Equation (6):

$$(\alpha h\nu)^p = A(h\nu - E_g) \quad (6)$$

where $h\nu$ is the incident photon energy, E_g the optical band gap energy, A is the constant depending on transition probability, and the power index p equals 2 for a direct allowed transition in optical absorption in MAPI [17]. E_g is calculated from the extrapolation of the linear part of the Kubelka-Munk plot.

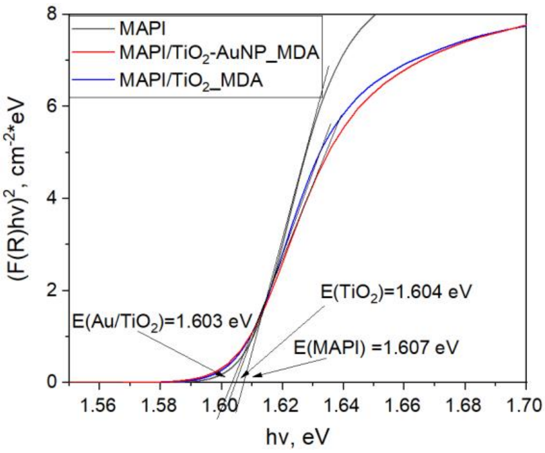

Figure S9. Plots of Kubelka-Munk function with calculated band gap values for 3 samples.

References

1. Keller, K.; Khramenkova, E.V.; Slabov, V.; Musin, A.; Kalashnikov, A.; Vinogradov, A.V.; Pidko, E.A. Inkjet Printing of Sc-Doped TiO_2 With Enhanced Photoactivity. *Coatings* **2019**, *9*, 78. https://doi.org/10.3390/coatings9020078.
2. Rubtsov, S.; Musin, A.; Zinigrad, M.; Kalashnikov, A.; Danchuk, V. New Strategy for Creating TiO_2 Thin Films with Embedded Au Nanoparticles. *Coatings* **2021**, *11*, 1525. https://doi.org/10.3390/coatings11121525
3. Danchuk, V.; Shatalov, N.; Pogreb, R.; Musin, A., Characterization of Sputtered ZnO Blocking Layers with Surface Plasmon Resonance Method. In Proceedings of the Seventeenth Russian-Israeli Bi-National Workshop, Ariel, Israel, 19–21 June 2019; pp. 186–193.
4. Li, P.; Jiang, X.; Huang, S.; Liu, Y.; Fu, N. Plasmonic Perovskite Solar Cells: An Overview from Metal Particle Structure to Device Design. *Surf Interfaces*, **2021**, *25*, 101287.
5. Farbod, M.; Khademalrasool, M.; Talebzadeh, M.D. Plasmon-Enhanced Photocatalysis Based on Ag Plasmonic Nanospheres and ZnO Nanoparticles: Synthesis and Study Mechanisms Governing the Plasmonic Photocatalytic Performance. *Plasmonics* **2016**, *12*, 759–769. https://doi.org/10.1007/s11468-016-0323-1.
6. Im, J.H.; Kim, H.S.; Park, N.G. Morphology-Photovoltaic Property Correlation in Perovskite Solar Cells: One-Step Versus Two-Step Deposition of $CH_3NH_3PBI_3$. *APL Mater*, **2014**, *2*, 081510.
7. Saliba, M.; Matsui, T.; Seo, J.Y.; Domanski, K.; Correa-Baena, J.P.; Nazeeruddin, M.K.; Zakeeruddin, S.M.; Tress, W.; Abate, A.; Hagfeldt, A. and et al. Cesium-Containing Triple Cation Perovskite Solar Cells: Improved Stability, Reproducibility and High Efficiency. *Energ. Environ. Sci.* **2016**, *9*, 1989–1997.
8. Luther, J.M.; Jain, P.K.; Ewers, T.; Alivisatos, A.P. Localized Surface Plasmon Resonances Arising from Free Carriers in Doped Quantum Dots. *Nat. Mater*, **2011**, *10*, 361–366.
9. Kheirandish, A.; Sepehri Javan, N.; Mohammadzadeh, H. Modified Drude Model for Small Gold Nanoparticles Surface Plasmon Resonance Based on the Role of Classical Confinement. *Sci. Rep.*, **2020**, *10*, https://doi.org/10.1038/s41598-020-63066-9
10. Jensen, T.R.; Duval, M.L.; Kelly, K.L.; Lazarides, A.A.; Schatz, G.C.; Van Duyne, R.P. Nanosphere Lithography: Effect of the External Dielectric Medium on the Surface Plasmon Resonance Spectrum of a Periodic Array of Silver Nanoparticles. *J. Phys. Chem. B*, **1999**, *103*, 9846–9853.
11. Workman, J.; Mark, J.H. Using Reference Materials, Part II: Photometric Standards. *Spectroscopy* **2019**, *34*.
12. Yuan, Z.; Wu, Z.; Bai, S.; Xia, Z.; Xu, W.; Song, T.; Wu, H.; Xu, L.; Si, J.; Jin, Y. and et al. Hot-Electron Injection in a Sandwiched TiOx-Au-TiOx Structure for High-Performance Planar Perovskite Solar Cells. *Adv. Energ. Mater*, **2015**, *5*, 1500038.
13. Macdonald, T.J.; Ambroz, F.; Batmunkh, M.; Li, Y.; Kim, D.; Contini, C.; Poduval, R.; Lui, H.; Shapter, J.G.; Papakonstaninou, I. and et al. TiO_2 Nanofiber Photoelectrochemical Cells Loaded with SUB-12 NM AuNPs: Size Dependent Performance Evaluation. *Mater. Today Energ*, **2018**, *9*, 254–263.
14. Luo, Q.; Zhang, C.; Deng, X.; Zhu, H.; Li, Z.; Wang, Z.; Chen, X.; Huang, S. Plasmonic Effects of Metallic Nanoparticles on Enhancing Performance of Perovskite Solar Cells. *Appl. Mater. Interfaces* **2017**, 9, 34821–34832.
15. Zhao, D.; Yu, M.; Zheng, L.; Li, M.; Dai, S.J.; Chen, D.; Lee, T.C.; Yun, D.Q. Enhanced Efficiency and Stability of Planar Perovskite Solar Cells Using a Dual Electron Transport Layer of Gold Nanoparticles Embedded in Anatase TiO_2 Films. *Appl. Energ. Mater.* **2020**, *3*, 9568–9575. https://doi.org/10.1021/acsaem.0c00276.
16. Simmons, E.L. Diffuse Reflectance Spectroscopy: A Comparison of the Theories. *Appl. Optics*, **1975**, *14*, 1380.
17. Kim, H.S.; Lee, C.R.; Im, J.H.; Lee, K.B.; Moehl, T.; Marchioro, A.; Moon, S.J.; Humphry-Baker, R.; Yum, J.H.; Moser, J.E. and et al. Lead Iodide Perovskite Sensitized All-Solid-State Submicron Thin Film Mesoscopic Solar Cell with Efficiency Exceeding 9%. *Sci. Rep.* **2012**, *2*, https://doi.org/10.1038/srep00591
